# Multiphase Simulated Annealing Based on Boltzmann and Bose-Einstein Distribution Applied to Protein Folding Problem

**DOI:** 10.1155/2016/7357123

**Published:** 2016-06-20

**Authors:** Juan Frausto-Solis, Ernesto Liñán-García, Juan Paulo Sánchez-Hernández, J. Javier González-Barbosa, Carlos González-Flores, Guadalupe Castilla-Valdez

**Affiliations:** ^1^Instituto Tecnológico de Ciudad Madero, Tecnológico Nacional de México, Avenida Sor Juana Inés de la Cruz s/n, Colonia los Mangos, 89440 Ciudad Madero, TAMPS, Mexico; ^2^Universidad Autónoma de Coahuila, Ciudad Universitaria, 25280 Arteaga, COAH, Mexico; ^3^UPEMOR, Boulevard Cuauhnáhuac 566, Jiutepec, 62550 Mor México, CP, Mexico

## Abstract

A new hybrid Multiphase Simulated Annealing Algorithm using Boltzmann and Bose-Einstein distributions (MPSABBE) is proposed. MPSABBE was designed for solving the Protein Folding Problem (PFP) instances. This new approach has four phases: (i) Multiquenching Phase (MQP), (ii) Boltzmann Annealing Phase (BAP), (iii) Bose-Einstein Annealing Phase (BEAP), and (iv) Dynamical Equilibrium Phase (DEP). BAP and BEAP are simulated annealing searching procedures based on Boltzmann and Bose-Einstein distributions, respectively. DEP is also a simulated annealing search procedure, which is applied at the final temperature of the fourth phase, which can be seen as a second Bose-Einstein phase. MQP is a search process that ranges from extremely high to high temperatures, applying a very fast cooling process, and is not very restrictive to accept new solutions. However, BAP and BEAP range from high to low and from low to very low temperatures, respectively. They are more restrictive for accepting new solutions. DEP uses a particular heuristic to detect the stochastic equilibrium by applying a least squares method during its execution. MPSABBE parameters are tuned with an analytical method, which considers the maximal and minimal deterioration of problem instances. MPSABBE was tested with several instances of PFP, showing that the use of both distributions is better than using only the Boltzmann distribution on the classical SA.

## 1. Introduction

In genetics DNA, RNA, and proteins are the basic elements for many researches. DNA is a molecule that contains genetic instructions, which are involved in protein synthesis process [1]. This molecule represents a complete set of hereditary information of any organism. DNA has four different nucleotides, which are adenine, cytosine, guanine, and thymine. This molecule is divided into genes, and a gene is a sequence of nucleotides that express a protein. A functional protein is conformed in an approximated geometrical model of the global minimum energy [2, 3]. This is a dinamic process where the lowest free energy of the protein plus the solvent can be reasonably approximated by the minimum free energy found by Monte Carlo, conformational space annealing, genetic algorithms, and some deterministic methods [3, 4]. In fact, there are some examples, such as insulin alphalytic [5, 6] with natural conformations whose energy is not minimal. This structure is usually named Native Structure (NS). In addition, the free energy of an NS conformation depends on the interaction among the atoms and their relative positions.

Protein Folding Problem (PFP) is an enormous challenge and important problem in bioinformatics, medicine, and other areas [7]. The function of a protein is directly related to its three-dimensional structure, and misfolded proteins can cause a variety of diseases. The aim of this problem is to find the natural tertiary structure of a protein using only a target sequence. A protein can take a high number of different conformational structures from its primary structure to its NS. The computational problem involved to find the NS is known as Protein Folding Problem. Because PFP is an NP-hard problem [8], heuristic methods avoiding the generation of all possible states of the protein are commonly used. In order to find an NS, computational methods search structures on a huge space of possible solutions. These methods can obtain several structures very close to the NS. A particular class of these methods is known to be* ab initio* which looks for the NS using only the protein's amino acid sequence.

As a consequence, to solve PFP, new metaheuristics are applied, where simulated annealing (SA) [9, 10] is one of the most successful [11–13]. Currently, classical SA applies a Boltzmann distribution in order to accept bad solutions and escape from local minima. However, to generate high-quality solutions for PFP, new and more efficient SA have been designed; one of them, named Chaotic Multiquenching Annealing Algorithm (CMQA), has obtained very good results for proteins such as Met^5^-enkephalin, proinsulin, T0549, T0335, and T0281 or 1PLXW, 1T0C, 2K5E, SR384, and 1A19, in PDB format, respectively. There are three central phases of this algorithm [14]: (i) Multiquenching Phase (MQP), (ii) Annealing Phase (AP), and (iii) Dynamical Equilibrium Phase (DEP). All of these phases are explained in the paper; for this introduction all we need to know is that each phase is designed with an annealing approach looking for finding the best configuration of the previous one. At the beginning of the process, MQP improves a random configuration through an annealing procedure executed at extremely very high temperatures; AP searches for a better solution than that of MQP with an annealing search applied at high temperatures, and, finally, DEP is applied at low temperatures looking for a better solution than that obtained by AP. As the classical SA, all of these phases apply Boltzmann distribution for accept bad solutions. However, Bose-Einstein distribution can also be used for escape from local minima [15]. Nevertheless, algorithms using these two distributions in different ranges of temperatures have not been published for PFP.

In this paper, a new SA algorithm named MPSABBE (Multiphase Simulated Annealing based on Boltzmann and Bose-Einstein distributions) is introduced. MPSABBE applies the Boltzmann and Bose-Einstein distributions at high and low temperatures, respectively. The paper shows that using both distributions the quality solution is improved. This paper is organized as follows. In Section 2, PFP is described. In Section 3, the classical SA and MPSABBE algorithms are explained. In Section 4, the SA applied for solving PFP is detailed. In Section 4, all the four MPSABBE's phases are presented. In Section 5, analytical tuning methods SA and MPSABBE are described. In Section 6, experimental results are shown. Finally, in Section 7, the conclusions of this research are discussed.

## 2. Protein Folding Problem

PFP is related to the questions of how and why a protein is folded into its NS. The proteins adopt an extreme number of possible conformations [16], which depends on the number of amino acids and the number of conformations by each amino acid. The essential concept introduced by Levinthal is that the PFP is a random search problem. This general idea means that all conformations of a protein (except the native state) are equally likely. Thus, it is more efficient to find the native state by a random search. PFP is an interdisciplinary problem that involves molecular biology, biophysics, computational biology, and computer science. In the* ab initio* case, NS prediction requires different mechanisms that lead the searching process to a biological three-dimensional structure. As was previously mentioned, this process requires only the amino acids' sequence. PFP is an enormous challenge and is very hard to find the NS of a protein because the space of possible conformations of the protein is in general extremely large. For all practical purposes, PFP can be defined as follows.

Given

(i) a sequence of *n* amino acids *a*_1_, *a*_2_,…, *a*_*n*_ that represents the primary structure of a protein,
(ii) an energy function *f* ^*∗*^(*σ*_1_, *σ*_2_,…, *σ*_*n*_), where the variables *σ*_1_, *σ*_2_,…, *σ*_*n*_ represent *n* dihedral angles,

find the following:

(i) the Native Structure such that *f* ^*∗*^(*σ*_1_, *σ*_2_,…, *σ*_*n*_) represents the lowest energy value, where
(ii) the solution *σ* ^*∗*^ = *σ*_1_, *σ*_2_,…, *σ*_*n*_ defines the best three-dimensional configuration.

Force fields are used to represent the energy of a protein; some of the most common are AMBER [17], CHARMM [18], ECEPP/2 [19–21], ECEPP/3 [22], and GROMACS [23]. These force fields compute energy components, for instance, the electrostatic energy, the torsion energy, the hydrogen bond energy, and the Lennard-Jones energy. In this paper ECEPP/2 force field is used.

The atoms of a protein are represented in three-dimensional cartesian coordinates. There are four types of torsion angles or dihedral angles as follows:

(i) The angle between the amino group and the alpha carbon is referred to as Phi (*ϕ*). This angle represents the angle between the amino group (or NH_2_) of the amino acid *i* and the alpha Carbon C_*i*_ in the sequence; specifically, it represents the bond angle between N_*i*_ atom of amino group and the central carbon (*α*C_*i*_).
(ii) The dihedral angle between the alpha carbon and the carboxyl group is referred to as Psi (*ψ*). Psi represents the angle between the carboxyl (COOH_*i*_) group of the amino acid *i* and the central carbon *i* (C_*i*_) of the same amino acid. In particular, Psi measures the angle of the covalent bond between C_*i*_ of the carboxyl group and the central carbon (*α*C_*i*_).
(iii) For every amino acids sequence, an omega angle (*ω*) is defined for each two consecutive amino acids *i* − 1, *i*; specifically, it is the angle of the covalent bond between the atom N_*i*_ of amino acid *i* and carbon C_*i*−1_ of the carboxyl group amino acid *i* − 1.
(iv) And, finally, each Chi angle (*χ*) is defined between the two planes conformed by two consecutive carbon atoms in the radical group.

The variables of the problem are all of these four angles which are in the range [0,360]. In the simulations conducted in this research work, these angles are set with discrete values. Some variables have well-defined ranges as is the case of Psi and Phi angles whose ranges are defined by the Ramachandran plot [24]. The Phi angle is defined in the ranges [180,300] and [45,60]. The Psi angle is defined in three ranges [20,180], [300,330], and [180,205]. Finally, the omega angle is fixed at 180 degrees.

## 3. Simulated Annealing Algorithm

### 3.1. Simulated Annealing Based on Boltzmann Distribution

Simulated Annealing (SA) Algorithm is a probabilistic method proposed by Kirkpatrick et al. [9] and Černý [10] and is an adaptation of the Metropolis algorithm, which is a Monte Carlo method [25]. SA is based on the gradual metal cooling for crystallization. This algorithm works by emulating the physical process where a metal is heating at very high temperature and then cooled very slowly until its frozen state. When this process happens, the metal is crystallized with the lowest energy configuration. SA is an algorithm that has been used for finding the optimal solution or close to it for different NP-hard problems including biological problems such as sequence alignment [26–28], phylogenetic trees [29], and PFP [30]. From a theoretical point of view, SA converges to the optimal solution or close to the lowest free energy [31]. However, classical SA is not able to find the lowest energy because energy barriers are too high for SA and cannot escape from local minima. As a consequence, variants of this method are proposed [14, 30].

Simulated annealing usually starts at a very high initial temperature (*T*_initial_). Through a cooling function, the temperature value is gradually reduced from *T*_initial_ to *T*_final_, which usually is very close to zero [9, 10]. There are several cooling functions used in SA [31–36], for example,

(1) Tk+1=αTk

(2) Tk+1=e−αTk

(3) Tk+1=Tk1+ηTk.

The most common function is (1). This function reduces the temperature parameter by *α* factor, which is commonly in the range of 0.7 ≤ *α* < 1.0. A slow cooling is applied when *α* is very close to 1, while a fast cooling is applied when *α* is around 0.70.

The classical SA has two cycles as is shown in Algorithm 1; the first one is named temperature cycle and is used to decrease the value of the temperature with a specific cooling function. The second cycle is named metropolis cycle and it generates, accepts, or rejects solutions of the problem to be optimized. The initial and final temperature values are set (see lines (1)-(2)). These values are obtained by an analytical (see Section 5) or experimental way: *T*_initial_ should be as high as possible, while *T*_final_ should be close to zero. An initial solution (*S*_initial_) is required in SA; this solution is generated (see line (3)) and is set to *S*_current_. At the beginning of the process, the parameter *T* is set at the initial temperature (see line (4)). The temperature cycle is executed from *T*_initial_ to *T*_final_ (see lines (5)–(19)). Then the metropolis cycle is repeated (see lines (6)–(17)) a certain number of times until a stop condition, which is explained later in this paper. A new solution (*S*_new_) is generated within the metropolis cycle by applying a small perturbation to the current solution *S*_current_ (see line (7)). The difference between these two solutions (*S*_new_ and *S*_current_) is calculated (see line (8)). In practice, SA can be stopped when the probability of accepting a new solution is negligible. For a minimization problem, if this difference is less than or equal to zero (see line (9)), the new solution is accepted (see line (10)). When this difference is greater than zero, the Boltzmann distribution is applied. Then, a Boltzmann probability is calculated using (4) in line (12). If this probability is higher than a random value between 0 and 1 (see line (13)), then the new solution *S*_new_ is accepted (see line (14)):

(4) $$\begin{array}{c} P\left(\;S_{new}\;\right)=e^{\left(-\Delta S/T\right)}. \end{array}$$

After the metropolis cycle is completed, the temperature value is reduced by a cooling function (see line (18)). For a maximization problem, if the difference of *S*_new_ − *S*_current_ is greater than zero, the new solution *S*_new_ is accepted; else *S*_new_ can be rejected or accepted depending on the Boltzmann probability value.

### 3.2. Simulated Annealing Based on Bose-Einstein Distribution

Statistical Mechanics (SM) study the overall behavior of a system consisting of a large number of particles whose behavior is unpredictable. SM uses statistics and probability theory and thermodynamic principles. According to SM, the occurrence of each future result is determined by a probabilistic function such as Boltzmann and Bose-Einstein distributions. In addition, only the most probable behavior of the system in thermal equilibrium at a given temperature is observed [37]. Bose-Einstein distribution is obtained by finding the most probable distribution, that is, solving the problem defined by maximizing the most probable distribution, subject to the following constraints: (*h*_1_) the number of particles (defined by the summation of particles in each microstate) is constant and (*h*_2_) the total energy (defined by the summation of individual energies of each microstate) is constant. The problem is solved using Lagrange multipliers. The parameters *λ* and *β* are defined as lagrage multiplier of *h*_1_ and *h*_2_, respectively [38]. Then the Bose-Einstein distribution applied for low and very low temperatures is defined by

(5) $$\begin{array}{c} h\left(\;\Delta E\;\right)=\frac{1}{\left(e^{\lambda +\beta_{}ei}-1\right)}. \end{array}$$

Then particles behavior can be modeled by Bose-Einstein distribution defined in (6). This equation defines the acceptance probability distribution of a new configuration of particles:

(6) $$\begin{array}{c} h\left(\;\Delta E\;\right)=\frac{1}{\left(e^{\lambda }e^{\left(\Delta E/KT\right)}-1\right)}, \end{array}$$

where *T* is the temperature parameter, *λ* is related to the constraint of the total of particles in the system, and *K* is the Boltzmann constant. However, at very high temperatures Bose-Einstein distribution practically becomes the Boltzmann distribution. Nevertheless, at low and very low temperatures, the particles behave differently and they tend to congregate at the same lowest energy state; the result is known as a Bose-Einstein condensate [39]; as a consequence, the system can be modeled by Bose-Einstein distribution. Section 4 presents a new SA applying both Boltzmann and Bose-Einstein distributions for accepting bad solutions for high and low temperatures.

### 3.3. Simulated Annealing Applied to Solve Protein Folding Problem

The classical Simulated Annealing Algorithm can be implemented to solve the Protein Folding Problem [40] as is shown in the pseudocode of Algorithm 2. The initial and final temperature (see lines (1)-(2)) can be calculated according to the instance of the problem by applying the analytical method parameters of Section 5; that means that the protein should be preprocessed.

Applying the cooling function (1), the cooling factor value *α* is required. The temperature value is reduced very slowly; thus, *α* must be very close to 1 (see line (3)). In order to reduce very fast the temperature, the cooling factor *α* is set very close to 0.70. An initial solution of PFP is created, which is set to the current solution *S*_current_ (see line (4)). The internal angles of the initial solution are modified at random. At this point, the best solution *S*_better_ is *S*_current_ (see line (5)). The energy of *S*_current_ is calculated by applying a force field function (see line (6)). Before starting the temperature cycle, the initial is loaded into *T* variable in line (7). Then the temperature cycle starts (see lines (8)–(26)) with a logic condition (*T* greater than *T*_final_ in line (8)). Inside of temperature cycle, the metropolis cycle is executed (see lines (9)–(24)). After this cycle is completed, the value of the temperature is decreased (see line (25)).

Inside the metropolis cycle, a new solution of Protein Folding Problem *S*_new_ is generated by modifying the previous solution *S*_current_. This is done by modifying the internal angles of the protein (see line (10)). The energy of the protein is calculated (see line (11)), and the difference of energies (i.e., between *S*_new_ and *S*_current_) is determined (see line (12)). This difference is denoted by Δ*S* = *S*_current_ − *S*_new_. The new solution is accepted when the new solution is better than the previous one; thus, the current solution *S*_current_ is replaced by *S*_new_ (see line (14)). When a new solution is worse than the current solution, it can be accepted using the Boltzmann distribution (see line (21)). The probability of this distribution (or acceptance probability) is directly related to the current value of the temperature and the difference of energy between *S*_new_ and *S*_current_. This probability is calculated by (4). As the temperature value is reduced, the acceptance probability *P*(*S*_new_) decreases.

## 4. MPSABBE Algorithm

### 4.1. General Description

**Figure 1:**
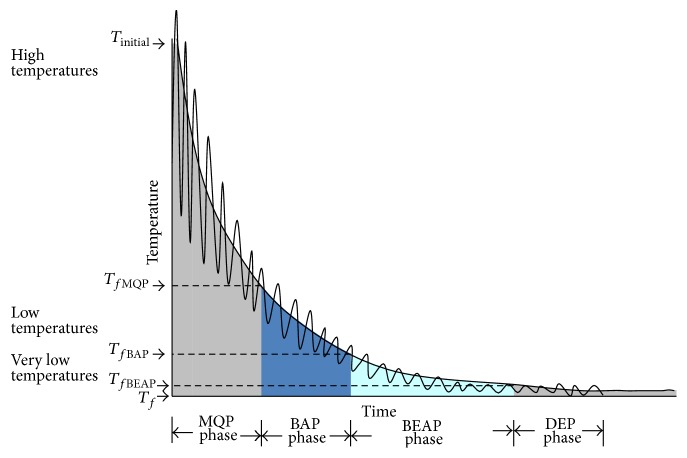
MPSABBE phases.

MPSABBE is a hybrid algorithm, which has four phases (see Figure 1). These phases are (i) Multiquenching Phase (MQP) applied from extremely high to high temperatures, (ii) Boltzmann Annealing Phase (BAP), which is executed from high to low temperatures, (iii) Bose-Einstein Annealing Phase (BEAP) from low to very low temperatures, and finally (iv) Dynamical Equilibrium Phase (DEP) which applies an annealing process at extremely low temperatures using Bose-Einstein distribution.

In order to accept worse solutions, BAP and BEAP apply Boltzmann and Bose-Einstein distributions, respectively. This is done with the aim of escaping from local minima. DEP is an extension of BEAP, where the stochastic equilibrium is dynamically detected. This is done by using a regression method into the metropolis cycle; the iterations' number is considered as the independent variable and the energy value of each iteration as the dependent variable. The equilibrium detection criterion is the slope of the energy function into the metropolis cycle. The four phases MQP, BAP, BEAP and DEP are executed in the temperatures range shown in Table 1. The initial and final temperatures *T*_initial_ and *T*_*f*_ are determined using the analytical tuning method of Section 6. The other temperatures are determined using a variability criterion, such as the variability being larger where the temperature is higher.

### 4.2. MQP Phase of MPSABBE

MQP has several subphases. It starts at an extremely high initial temperature (*T*_initial_), which is obtained by an analytical method [41]. This phase is finished when a threshold temperature (*T*_*f*MQP_) is reached. MQP uses the cooling function given by

(7) $$\begin{array}{c} T_{k+1}=\alpha_{Quenching}\gamma_{k}T_{k}, \end{array}$$

where *α*_Quenching_ is a decrement factor of the temperature parameter, in the range [0.7,1.0], and defines how fast each MQP subphase is decreased. A very low *α*_Quenching_ value will decrease the temperature very fast. Besides, *γ*_*k*_ is defined as

(8) $$\begin{array}{c} \gamma_{k}=1-\tau_{k}. \end{array}$$

The *τ* parameter is defined by (9), where 0 < *τ* < 1, and it defines a quadratic decrement of the temperature. Notice that *τ* converges to zero and (7) is equivalent to (10):

(9) τk=τk−12

(10) Tk+1=αQuenchingTk.

**Figure 2:**
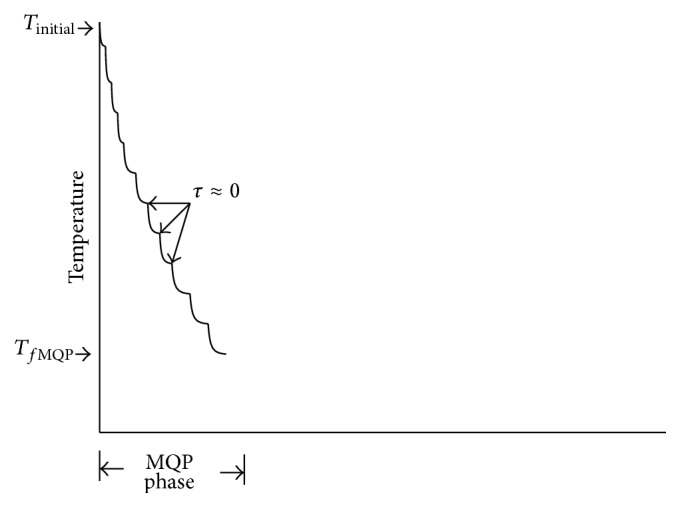
MQP phase of MPSABBE algorithm.

The transition between two subphases is based on *τ* parameter; it occurs when *τ* converges to zero (*τ* ≈ 0). When *τ* is very close to zero, a new MQP subphase is started and *τ* is set to its initial value. This process continues until the temperature *T*_*f*MQP_ is reached. In Figure 2, the MQP phase is shown. In this phase, several subphases are shown. When a subphase is started, the parameter *τ* is set to its initial value.

In Algorithm 3, the MQP pseudocode of MPSABBE is shown. At setting section (see lines (4)–(6)), the initial temperature is calculated by an analytical method. The final temperature of this phase (*T*_*f*MQP_) is set to an initial value, determined in an experimental way. In line five, the variable *T* is set to the initial temperature. The factors *α*_Quenching_ and *τ* are set to their initial values. The initial solution *S*_current_ is generated (see line (8)). The energy of this solution Energy(*S*_current_) is calculated, and *E*(*S*_current_) and *S*_min_ are set to Energy(*S*_current_) and *S*_current_, respectively.

The external cycle is started at line (10), and this is finished at line (31). This internal cycle generates solutions of PFP and accepts or rejects solutions using the Boltzmann distribution. The temperature parameter is decreased into this cycle by applying a cooling function (see line (30)). In this cycle, *τ* is set by (9) (see line (26)). When *τ* is very close to zero, this variable is set to its initial value (see line (28)). The Temperature value is calculated by (7).

After the external cycle is started, the metropolis cycle is started too. This cycle generates new solutions of PFP. A new solution *S*_new_ is obtained by applying a small perturbation to the current solution *S*_current_ (see line (12)). The difference between the energies of *S*_new_ and *S*_current_ is calculated (see line (13)). If this difference is less than zero (see line (14)), then the new solution *S*_new_ is accepted. *S*_current_ is replaced by *S*_new_ (see line (15)). *E*(*S*_current_) is replaced by *E*(*S*_new_) (see line (16)). If the difference of energies between these solutions is larger than zero, then the Boltzmann probability is applied (see line (17)). If this probability is larger than a random number between 0 and 1 (see line (17)), then the new solution *S*_new_ is accepted (see line (18)). The *S*_current_ is replaced by *S*_new_ (see line (19)). If *E*(*S*_current_) is less than *E*(*S*_new_) (see line (21)) then *S*_min_ is set to *S*_current_ (see line (22)). The *E*(*S*_min_) is replaced by *E*(*S*_current_) (see line (23)).

### 4.3. BAP Phase of MPSABBE

In Algorithm 4, pseudocode of BAP is shown. BAP is based on simulated annealing. The temperature parameter is decreased by (*T*_*k*+1_ = *α*_Annealing_ *T*_*k*_) or (*T*_*k*+1_ = *e* ^−*α*_Annealing_^ *T*_*k*_). On the other hand, the length of metropolis cycle is determined by (21) or (27), respectively. In the internal cycle of the BAP, new solutions for the instance are generated. In this cycle, a better solution than a previous one is always accepted. However, worse solutions are accepted or rejected by applying the Boltzmann distribution (4). The length of the Markov chain (i.e., the internal cycle length) is determined by (21), where the increment *β* is calculated with (22). The initial temperature was set to a threshold value, which was the final temperature of MQP phase. The final temperature of BAP phase is very close to zero.

### 4.4. BEAP Phase of MPSABBE

In Algorithm 5, pseudocode of BEAP is shown. Again the external cycle decreases its temperature value according to the cooling functions (1) or (2). This time, the metropolis cycle length is constant, and it is equal to the maximum length of the last metropolis cycle in BAP phase. In this second cycle, the Bose-Einstein distribution is applied for accepting worse solutions.

### 4.5. DEP Phase of MPSABBE

In Algorithm 6, the DEP goal is to detect the stochastic equilibrium by determining the iteration where the slope of the energy function remains very close to zero. In order to do that, let us define the next variables: (a) *x*_*i*_ the number of the iterations in the metropolis cycle (1,2,…, *n*) and (b) *E*_*i*_ the energy found for the algorithm in iteration *x*_*i*_. Using a standard least squares method, the slope for *n* iterations is defined by

(11) $$\begin{array}{c} m=\frac{n\sum_{i=1}^{n}x_{i}E_{i}-\left(\sum_{i=1}^{n}x_{i}\right)\left(\sum_{i=1}^{n}E_{i}\right)}{n\sum_{i=1}^{n}x_{i}^{2}-{\left(\sum_{i=1}^{n}x_{i}\right)}^{2}}, \end{array}$$

which becomes

(12) $$\begin{array}{c} m=k_{1}\overset{n}{\underset{i=1}{\sum }}iE_{i}-k_{2}\overset{n}{\underset{i=1}{\sum }}E_{i}, \end{array}$$

where

(13) k1=12n3−n,k2=6n2+n.

Notice that the complexity of the computation of (12) is *O*(*n*). This equation contains only summations; thus, it is less complex than (11). These summations are computed using simple data structures. *k*_1_ and *k*_2_ are only constants for a particular *n* value.

## 5. Analytical Tuning Method

### 5.1. Parameters Setting Based on Boltzmann Distribution

Parameters of MPSABBE are tuned by the analytical method [42]. The initial temperature is defined by the maximum difference named maximum decrement Δ*Z*_max_, which is calculated using a sample of random protein structures at the highest temperature range. In this sample, the energy of two consecutive protein structures defines a simple decrement of energy Δ*Z*_*i*,*j*_, and Δ*Z*_max_ is the maximum difference in the sample. On the other hand, the final temperature is calculated by applying the minimum deterioration (i.e., minimum decrement) Δ*Z*_min_ of a sample of protein structures taken at low temperatures. Analytical tuning based on Boltzmann distribution can be helpful for setting up the initial temperature. The probability of accepting any new solution *S*_new_ is near to one (*P*(*S*_new_) ≈ 1) at high temperatures, so the decrement of the cost function is maximal. The initial temperature (*T*_initial_) is associated with the maximum deterioration admitted and the defined acceptance probability *P*(*S*_new_).

Let *S*_current_ be the current solution and *S*_new_ a new proposed one, and *Z*(*S*_current_) and *Z*(*S*_new_) are the costs associated to *S*_current_ and *S*_new_, respectively. The maximum and minimum deteriorations are Δ*Z*_max_ and Δ*Z*_min_, respectively; then *P*(Δ*Z*_max_) probability of accepting a new solution *S*_new_ with the maximum deterioration is defined by

(14) $$\begin{array}{c} P\left(\;\Delta Z\;\right)=\exp\left(\;\frac{-\Delta Z}{T}\;\right). \end{array}$$

This equation is basically the Boltzmann distribution, which is applied for calculating *T*_initial_. This temperature value is defined by

(15) $$\begin{array}{c} T_{initial}=\frac{-\Delta Z_{max}}{\ln\left(P\left(\Delta Z_{max}\right)\right)}. \end{array}$$

Similarly, the final temperature (*T*_final_) is established according to the probability of accepting a new solution *S*_new_ with the minimum deterioration. The equation for calculating the final temperature is defined by

(16) $$\begin{array}{c} T_{final}=\frac{-\Delta Z_{min}}{\ln\left(P\left(\Delta Z_{min}\right)\right)}. \end{array}$$

There are other parameters of MPSABBE that are calculated by applying a particular cooling function; for example, the metropolis cycle length is calculated by applying

(17) $$\begin{array}{c} T_{k+1}=\alpha T_{k}. \end{array}$$

The analytical method determines the metropolis cycle length *L*_*k*_ with a simple Markov model [42]; at high temperatures, only a few iterations are required because, in this condition, the stochastic equilibrium is reached very fast. Nevertheless, at low temperatures, a more exhaustive exploration is needed and *L*_*k*_ should be as largest as possible. Let *L*_1_ be *L*_*k*_ at the temperature *T*_initial_ and let *L*_max_ be the maximum metropolis cycle length. Let the temperature *T*_*k*_ be decreased by the cooling function (17) and let *L*_*k*+1_ be calculated by

(18) $$\begin{array}{c} L_{k+1}=\beta L_{k}, \end{array}$$

where *β* is the increment coefficient of metropolis cycle (*β* > 1), so *L*_*k*+1_ > *L*_*k*_ and *L*_1_ is the initial value. The markov chain length of the last metropolis cycle is equal to *L*_max_. Functions (17) and (18) are consecutively applied in simulated annealing from *T*_initial_ to *T*_final_; consequently *T*_*n*_ and *L*_max_ are obtained by (19) and (20), respectively,

(19) Tn=αnTinitial

(20) Lmax=βnL1,

where *n* is the steps number from *T*_initial_ to *T*_final_.

Notice that the increment coefficient *β* can be calculated if the initial length *L*_1_ and the maximum length value *L*_max_ are available. As is well known the former can simply be set close to one, while the second depends on the exploration level established in the algorithm as follows.

Thus, the number of times that the metropolis cycle is executed can be simply obtained by using (21). Once *n* is determined the increment of the metropolis cycle length can be calculated by (22):

(21) n=ln⁡Tfinal−ln⁡Tinitialln⁡α

(22) β=exp⁡ln⁡Lmax−ln⁡L1n.

### 5.2. Parameters Setting Based on Bose-Einstein Distribution

The initial and final temperatures can be calculated by applying the Bose-Einstein distribution. Then, the probability of accepting a new solution with the maximum deterioration *P*(Δ*Z*_max_) is defined by (23). Consequently, the initial and final temperatures are calculated with (24) and (25), respectively,

(23) PΔZ=1eΔZ/T−1

(24) Tinitial=ΔZmaxln⁡PΔZmax+1/PΔZmax

(25) Tfinal=ΔZminln⁡PΔZmin+1/PΔZmin.

Let *T*_*k*_ be decreased by the cooling function (2). Thus, *T*_*n*_ is calculated by

(26) $$\begin{array}{c} T_{n}=e^{-n\alpha }T_{initial}. \end{array}$$

As a consequence, *n* and *β* are calculted by

(27) n=ln⁡Tfinal−ln⁡Tinitial−α

(28) β=exp⁡ln⁡Lmax−ln⁡L1n.

Notice that the increment coefficient *β* can be calculated if the initial and maximum metropolis length *L*_1_ and *L*_max_ are available [42]. As is well known the former can simply be set close to one, while the second depends on the exploration level established in the algorithm. Therefore, for any *S*_*i*_ solution, the value of *L*_max_ depends on the size of neighborhood |*V*_*si*_|. Thus, *L*_max_ = *C*|*V*_*si*_| and *C* = −ln⁡(*P*_*r*_(*S*_*i*_)), where *P*_*r*_(*S*_*i*_) is the rejection probability for a solution *S*_*i*_. The parameter *C* ranges from 1 to 4.6; the larger value of *C* assures a good exploration level in the neighborhood of *S*_*i*_ at the final temperature. Hence, different exploration levels can be applied. When we explore with *P*_*r*_(*S*_*i*_) values of 63%, 86%, 95%, or 99%, the exploration levels are *C* = 1, 2, 3, or 4.6, respectively. Because *L*_max_ can be very large for PFP instances, it is important to apply a particular process for detecting the stochastic equilibrium; this is done in DEP phase of MPSABBE that detects efficiently the stochastic equilibrium. The next section explains all MPSABBE phases and the performance of using Boltzmann and Bose-Einstein distribution.

## 6. Experimental Results

MPSABBE is tested with five instances of PFP, which are Met^5^-enkephalin, proinsulin, T0549, T0335, and T0281. These instances have different sequence's length and a different number of variables (dihedral angles). The smallest sequence is Met^5^-enkephalin, which has five amino acids and 19 variables. The largest sequence is a hypothetical protein (CASP T0281), which has 90 amino acids and 458 variables. The proinsulin instance has 31 amino acids and 132 variables; the 2K5E (CASP T0549) has 73 amino acids and 343 variables. The instance* Bacillus subtilis* (CASP T0335) has 85 amino acids and 450 variables. The dihedral angles used in the simulations were phi (Φ), psi (Ψ), omega (*ω*), and Chi (*χ*). The initial and final temperature are tuned analytically. In MQP, parameters *α*_Quenching_ and *τ* are set with 0.85 and 0.999, respectively. In each subphase of MQP the final value of *τ* is set to 0.001.

**Figure 3:**
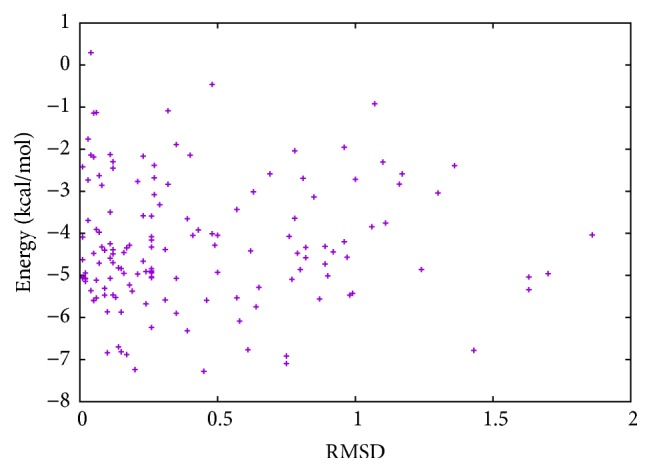
Energy and RMSD for Met^5^-enkephalin.

**Figure 4:**
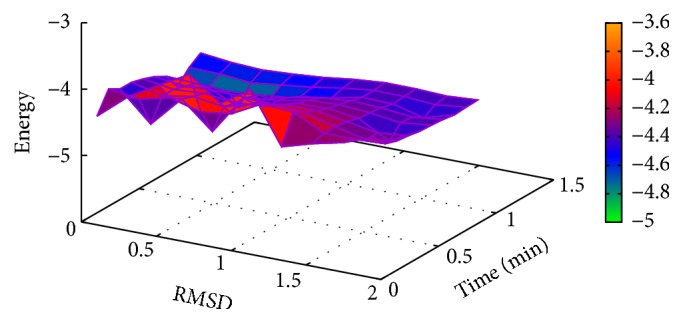
Landscape of energy, RMSD, and processing time for Met^5^-enkephalin.

In Table 2, the results of Met^5^-enkephalin obtained with MPSABBE algorithm are shown. In this table, we show the traditional average energy, processing time in minutes, and the average of the traditional RMSD (Root-Mean-Square Deviation) [43]. The RMSD was calculated using TM-Align [44]. The best average solution for Met^5^-enkephalin is −5.0634 kcal/mol with 0.8427 minutes of processing time, and the average RMSD obtained was 0.361 Å (Angstroms). The RMSD is a measure which represents a structural alignment between two proteins (target and solution). The target used in this paper was taken from Protein Data Bank (PDB). An RMSD near to zero is taken as a perfect structural alignment between both proteins. The RMSD is commonly used in protein folding to represent how a new obtained solution by simulation is structurally similar to the target solution. In this case, in Figure 3, the graphic of energy and RMSD for each solution is shown. In this graphic, all energies of Met^5^-enkephalin calculated by MPSABBE are plotted. This is a solution with poor quality because there are better solutions in the literature; the energy found by MPSABBE was −7.2787 kcal/mol. In Figure 4, the graphics of landscape of Met^5^-enkephalin is shown. The results obtained in the literature for this case by using ECEPP/2 and with *ω* fixed at 180 or *ω* variable were −10.72 [20] and −12.90 [43, 45], respectively. Examining the features of MPSABBE the exploration ability is not good enough; thus, the algorithm requires improvement. Figure 3 shows all solutions generated by MPSABBE; the curve enveloping the number of solutions in Figure 3 is only a descriptive tool to illustrate that the optimal solution is reached when the RMSD is too small; however, this is not really a very good stop condition. Notice that the best result obtained with the classical simulated annealing in the literature using Boltzmann distribution was only −5 kcal/mol [43], while the best result obtained in this case for MPSABBE using Bose-Einstein distribution was −7.2787 kcal/mol.

**Figure 5:**
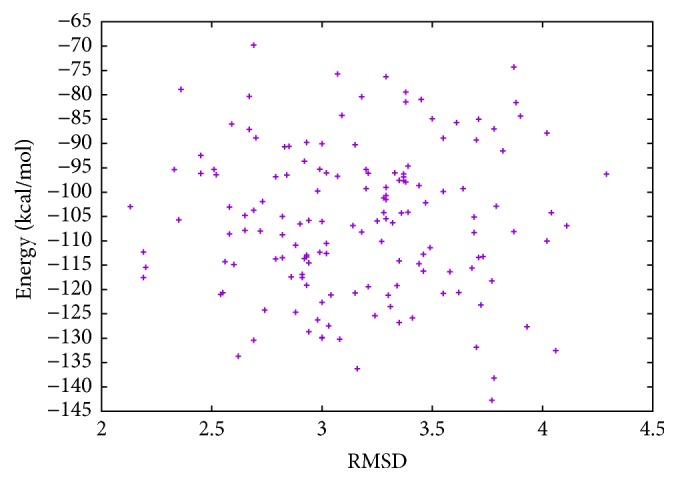
Energy and RMSD of proinsulin.

**Figure 6:**
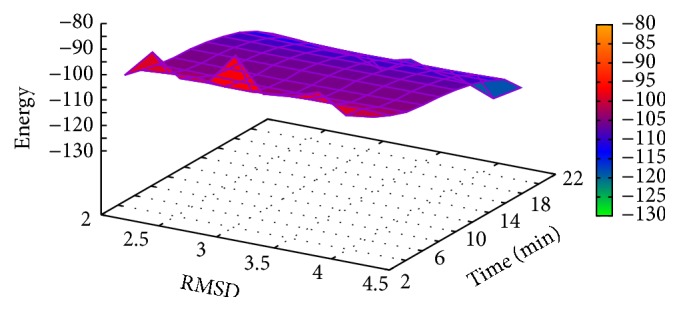
Landscape of energy, RMSD, and processing time for proinsulin.

In Table 3, the results of proinsulin obtained with MPSABBE algorithm are shown. The best average solution for this instance is −122.4350 kcal/mol with 20.7302 minutes of processing time, the average RMSD is 3.127 Å. This solution was obtained with *α*_Annealing_ = 0.95. In Figure 5 the graphic of energy and RMSD for each solution is shown. In this Figure, some energies of proinsulin calculated by MPSABBE are plotted. The best solution found by MPSABBE was −142.7586 kcal/mol. In Figure 6, the landscape of proinsulin is shown.

**Figure 7:**
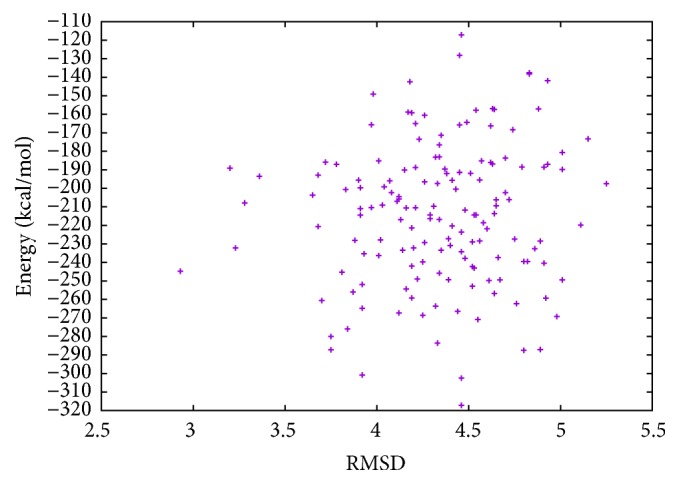
Energy and RMSD for T0549.

**Figure 8:**
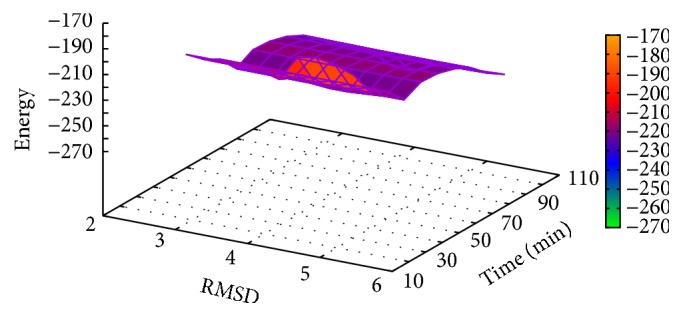
Landscape of energy, RMSD, and processing time for T0549.

In Table 4, the results of T0549 instance obtained with MPSABBE algorithm are shown. The best average solution for this instance is −257.0625 kcal/mol with 106.6151 minutes of processing time, the average RMSD is 4.30 Å. This solution was obtained with *α*_Annealing_ = 0.95. In Figure 7, the energy and RMSD for each solution are shown. In this figure, some energies of T0549 instance calculated by MPSABBE are plotted. The best solution found was −317.2117 kcal/mol. In Figure 8, the landscape of T0549 is shown.

**Figure 9:**
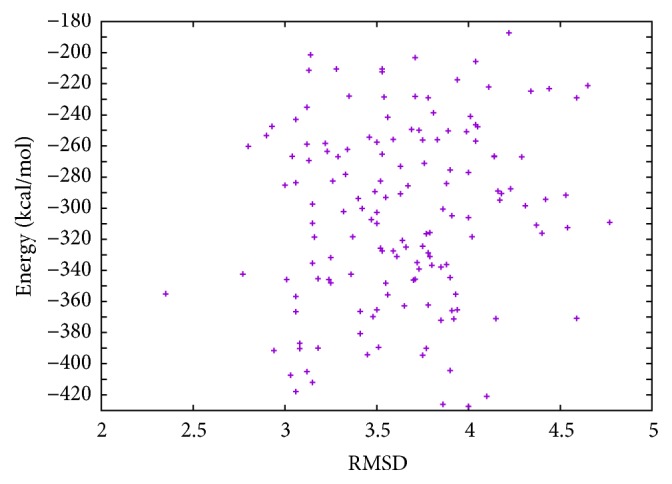
Energy and RMSD of T0335.

**Figure 10:**
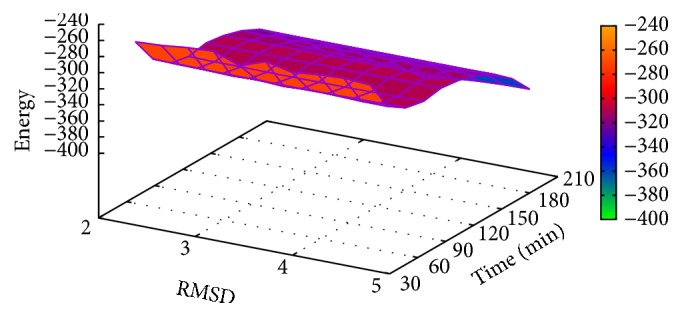
Landscape of energy, RMSD, and processing time of T0335.

In Table 5, the results of T0335 instance obtained with MPSABBE algorithm are shown. The best average solution for this instance is −378.6827 kcal/mol with 202.2453 minutes of processing time; the average RMSD is 3.5793. This solution was obtained with *α*_Annealing_ = 0.95. In Figure 9, the energy and RMSD for each solution are shown. In this figure, some energies of T0335 instance calculated by MPSABBE are plotted. The best solution was −427.2939 kcal/mol. In Figure 10, the landscape of T0335 is shown.

**Figure 11:**
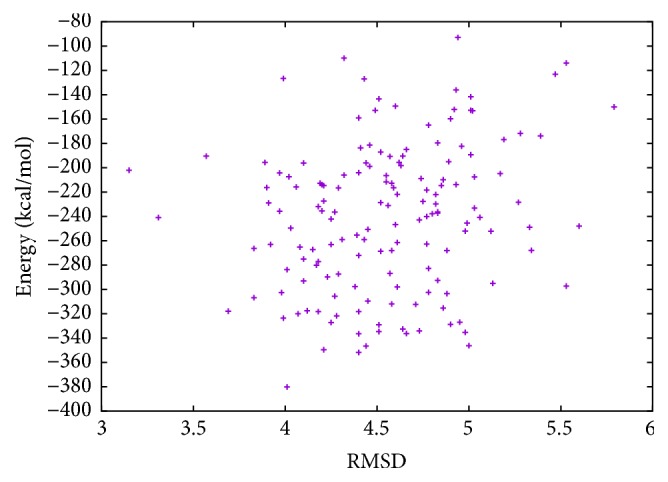
Energy and RMSD for T0281.

**Figure 12:**
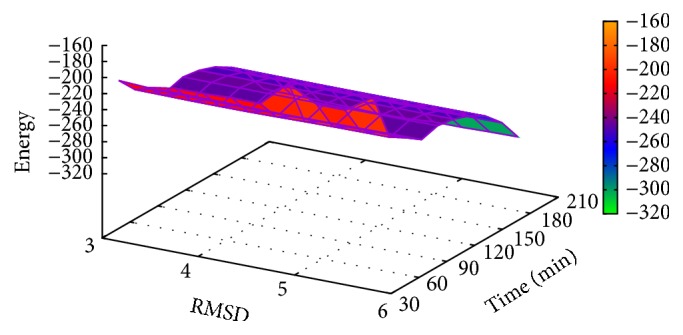
Landscape of energy, RMSD, and processing time for T0281.

In Table 6, the results of T0281 instance obtained with MPSABBE algorithm are shown. The best average solution for this instance is −322.3821 kcal/mol with 187.5070 minutes of processing time; the average RMSD is 4.5 Å. This solution was obtained with *α*_Annealing_ = 0.95. In Figure 11, the graphic of energy and RMSD for each solution are shown. In this figure, some energies of T0281 instance calculated by MPSABBE are plotted. The best solution found was −380.1765 kcal/mol. In Figure 12, the landscape of T0281 is shown.

**Figure 13:**
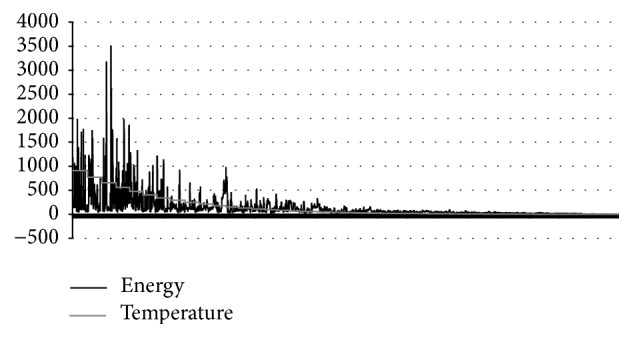
Energy of MPSABBE with Met^5^-enkephalin instance.

**Figure 14 fig14:**
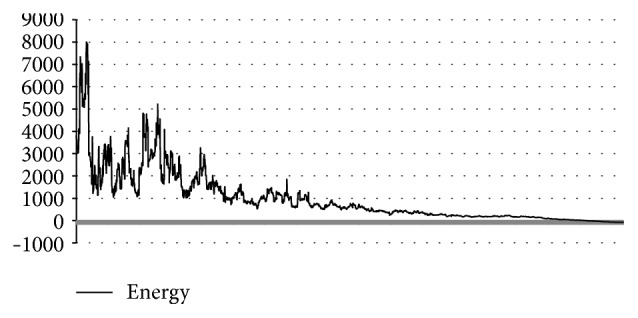
Energy of MPSABBE with proinsulin instance.

**Figure 15:**
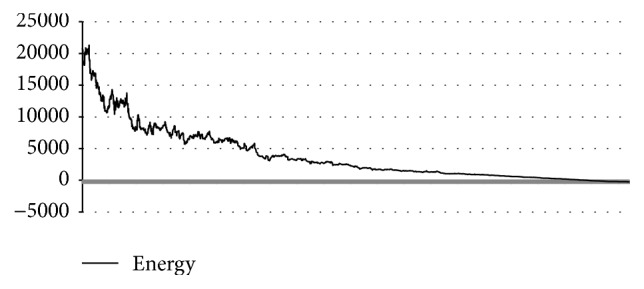
Energy of MPSABBE with T0281 instance.

Figures 13 [Fig fig14]–15 show the graphs of energy, which are obtained from consecutive solutions in the cycle of metropolis in specific executions. These figures correspond to the results of energies obtained from the MPSABBE algorithm with Met^5^-enkephalin, proinsulin, and T0281 instances, respectively.

### 6.1. Test Hypothesis

In Table 7, the average and deviation of energy and time for each instance applying MPSABBE algorithm are shown. The null hypothesis is defined as *H*_0_ : *μ*_*Q*MPSABBE_ ≤ *μ*_*Q*CMQA_, which means that the average energy of MPSABBE (*μ*_*Q*MPSABBE_) for each instance is less than or equal to CMQA (*μ*_*Q*CMQA_) [14]. The alternative hypothesis is defined as *H*_1_ : *μ*_*Q*MPSABBE_ > *μ*_*Q*CMQA_. In Table 8, the average and standard deviation of energy and time for each instance applying the proposed algorithm are shown. The average processing times are used for testing the null hypothesis, which is defined as *H*_0_ : *μ*_*T*MPSABBE_ ≤ *μ*_*T*CMQA_, which means that the average processing time of MPSABBE (*μ*_*T*MPSABBE_) is less than or equal to the average processing time of CMQA (*μ*_*T*CMQA_). The alternative hypothesis is defined as *H*_1_ : *μ*_*T*MPSABBE_ > *μ*_*T*CMQA_. In Table 9, the values obtained for *t*-student are shown; these values were calculated by applying the average and standard deviation of energy and execution time from Tables 7 and 8.

The value of *t*-student is −2.6363 (Table 9). The critical value is 1.645. The statistic test determined that the null hypothesis is accepted; thus, MPSABBE generates better quality solution than CMQA, when these approaches are applied with Met^5^-enkephalin instance. Therefore, the null hypothesis *H*_0_ : *μ*_*Q*MPSABBE_ ≤ *μ*_*Q*CMQA_ is rejected, and the average energy of MPSABBE (*μ*_*Q*MPSABBE_) for Met^5^-enkephalin instance is less than or equal to CMQA (*μ*_*Q*CMQA_). For processing execution time, the value of the statistic test (*t*-student) is −2.4022. Thus, MPSABBE (applied to Met^5^-enkephalin instance) uses less processing execution time than CMQA.

When the proinsulin instance is applied, the value of the statistic test (*t*-student) is −0.4272; thus, MPSABBE generates better quality solution than CMQA. For processing execution time, the value of the statistic test (*t*-student) is −3.0368. MPSABBE (applied to proinsulin instance) uses less processing execution time than the average processing time of CMQA. When the T0549 instance is applied, the value of the statistic test (*t*-student) is 0.3522, so that MPSABBE generates better quality solution than CMQA. For processing time, the value of the statistic test (*t*-student) is −4.0444. The MPSABBE (applied to T0549 instance) uses less processing execution time than CMQA. When the T0335 instance is applied, the value of the statistic test (*t*-student) is 0.5573, so that MPSABBE generates better quality solution than CMQA. For the processing execution time, the value of the statistic test (*t*-student) is −3.5832. The MPSABBE (applied to T0335 instance) uses less processing time than CMQA. When the T0281 instance is applied, the value of the statistic test (*t*-student) is 1.0515; thus, MPSABBE generates better quality solution than CMQA. For processing execution time test, the value of the statistic test (*t*-student) is −4.0533. Then MPSABBE (applied to T0281 instance) uses less processing execution time than CMQA. Therefore, MPSABBE generates the better quality solution and uses less processing execution time than CMQA in all instances.

Notice that the improvement obtained when the two distributions are used is better when the protein is smaller. For instance, for Met^5^-enkephalin and proinsulin (with five and thirty-one amino acids) MPSABBE surpass CMQA by 13.73 and 1.1243%, respectively; otherwise for T0549, T0335, and T0281 (with 73, 85, and 90 amino acids), these figures were −1.12, −2.13, and −3.75%, respectively. Thus, the new algorithm obtains better results for small proteins than the classical SA.

## 7. Conclusions

In this paper, a new Simulated Annealing Algorithm named MPSABBE for Protein Folding Problem is presented. This algorithm includes Bose-Einstein and Boltzmann distributions in SA. Traditionally, for PFP, SA only uses the Boltzmann distribution function as the acceptance probability of bad solutions. MPSABBE was compared to a classical SA for protein folding which only applies Boltzmann distribution. According to the experimentation, the new algorithm is more efficient by the use of the two distributions when the proteins are small. The quality of the solutions obtained by the new approach is not always the best alternative, although the difference of the quality solution is only 2 to 5% for the worse cases. Besides, the new approach can overtake the classical quality solution of SA by one to ten percent while execution time is in general lower.

## Figures and Tables

**Algorithm 1:**
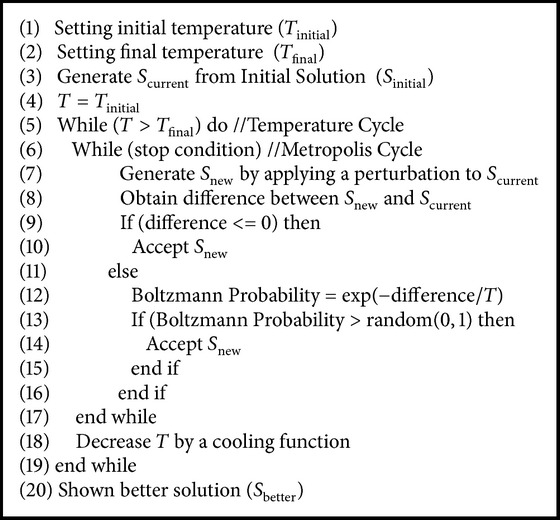
Pseudocode of classical simulated annealing.

**Algorithm 2:**
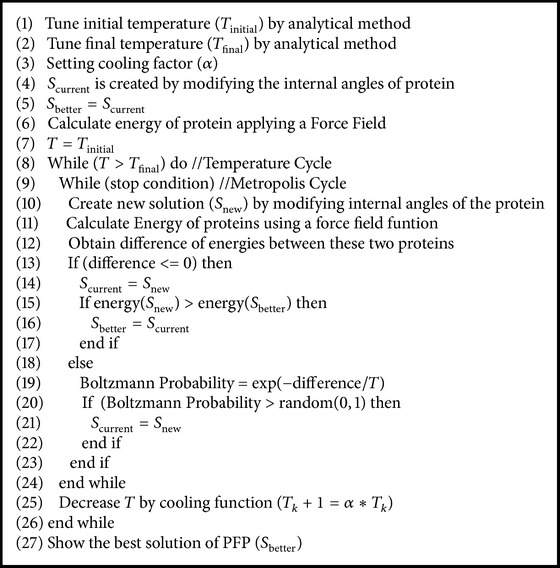
Pseudocode SA applied to protein folding problem.

**Algorithm 3:**
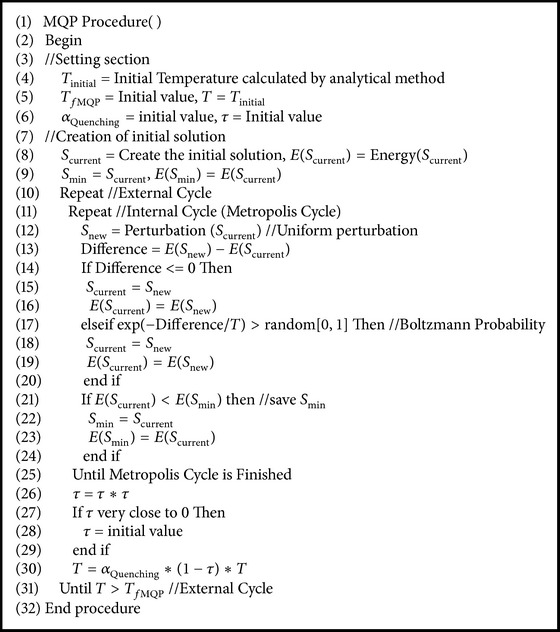
MQP pseudocode of MPSABBE algorithm.

**Algorithm 4:**
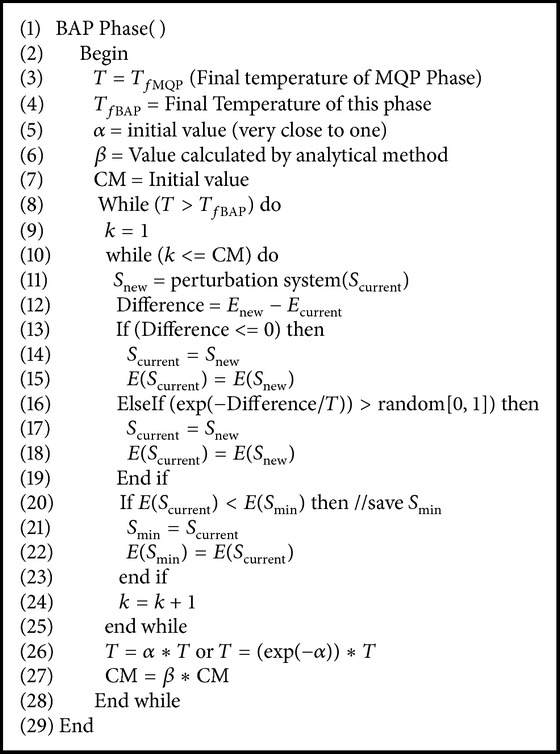
Pseudocode of BAP phase of MPSABBE.

**Algorithm 5:**
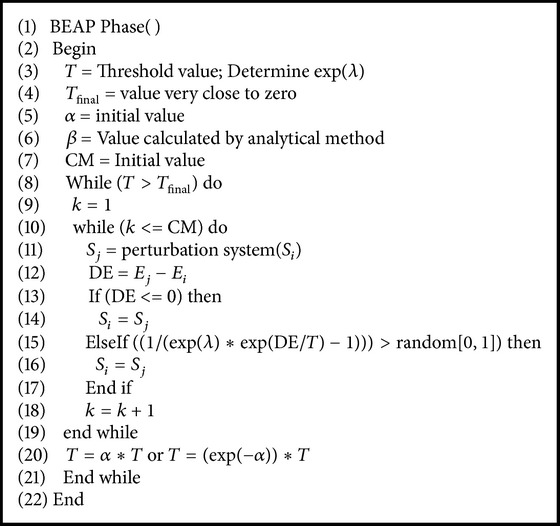
Pseudocode of BEAP phase.

**Algorithm 6:**
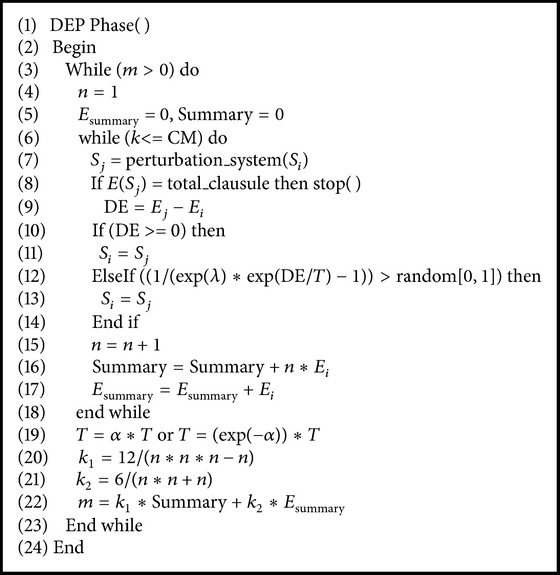
Pseudocode of DEP phase.

**Table 1:** Temperatures ranges of MPSABBE.

Phase	Initial temperature	Final temperature
MQP (from very high to high temperatures)	*T*_initial_	*T*_*f*MQP_
BAP (from high to low temperatures)	*T*_*f*MQP_	*T*_*f*BAP_
BEAP (from low to very low temperatures)	*T*_*f*BAP_	*T*_*f*BEAP_
DEP (from very low to extremely low temperatures)	*T*_*f*BEAP_	*T*_*f*_

**Table 2:** Average results of Met^5^-enkephalin with MPSABBE algorithm.

*α*_Annealing_	Average energy (kcal/mol)	Processing time (minutes)	Average RMSD (Å)
0.75	−3.0836	0.1252	0.4517
0.80	−4.3025	0.1701	0.4327
0.85	−4.4093	0.2023	0.3510
0.90	−4.6493	0.3384	0.5097
0.95	−5.0634	0.8427	0.3610

**Table 3:** Average results of proinsulin with MPSABBE algorithm.

*α*_Annealing_	Average energy (kcal/mol)	Processing time (minutes)	Average RMSD (Å)
0.75	−94.2520	3.0279	3.1370
0.80	−102.5484	3.8918	3.1153
0.85	−102.1247	5.1319	3.1253
0.90	−108.1093	7.8184	3.3083
0.95	−122.4350	20.7302	3.1273

**Table 4:** Average results of T0549 with MPSABBE algorithm.

*α*_Annealing_	Average energy (kcal/mol)	Processing time (minutes)	Average RMSD (Å)
0.75	−183.6351	19.4805	4.3933
0.80	−190.2890	24.9117	4.4180
0.85	−208.0338	31.1958	4.2933
0.90	−231.2849	48.6717	4.2887
0.95	−257.0625	106.6151	4.3037

**Table 5:** Average results of T0335 with MPSABBE algorithm.

*α*_Annealing_	Average energy (kcal/mol)	Processing time (minutes)	Average RMSD (Å)
0.75	−249.4399	32.9611	3.7413
0.80	−267.4245	40.4676	3.6750
0.85	−293.0409	52.2383	3.6160
0.90	−335.0567	78.9619	3.5828
0.95	−378.6827	202.2453	3.5793

**Table 6:** Average results of T0281 with MPSABBE algorithm.

*α*_Annealing_	Average energy (kcal/mol)	Processing time (minutes)	Average RMSD (Å)
0.75	−188.9717	32.7761	4.6160
0.80	−193.9981	40.4018	4.6347
0.85	−236.3011	53.3635	4.5507
0.90	−263.1571	79.3565	4.4467
0.95	−322.3821	187.5070	4.5515

**Table 7:** Average of energy and standard deviation of MPSABBE.

Instance	Energy average (kcal/mol)	Energy standard deviation	Time average (minutes)	Time standard deviation
Met^5^-enkephalin	−4.3016	0.7410	0.3357	0.2943
Proinsulin	−105.8938	10.4815	8.8670	8.1788
T0549	−214.0610	30.3024	46.1749	35.5258
T0335	−304.7289	52.3785	91.6016	76.1357
T0281	−240.9620	54.8912	85.0103	71.3112

**Table 8:** Average of energy and standard deviation of CMQA.

Instance	Energy average (kcal/mol)	Energy standard deviation	Time average (minutes)	Time standard deviation
Met^5^-enkephalin	−3.7820	0.7848	0.5719	0.4509
Proinsulin	−104.7165	10.8593	18.9617	16.2658
T0549	−217.1220	36.7019	121.9018	96.2037
T0335	−311.3921	39.3025	204.2191	154.3906
T0281	−254.3024	42.6025	231.8738	185.2004

**Table 9:** *t*-student for each instance.

Instance	*t*-student (for energy)	*t*-student (for time execution)
Met^5^-enkephalin	−2.6363	−2.4022
Proinsulin	−0.4272	−3.0368
T0549	0.3522	−4.0444
T0335	0.5573	−3.5832
T0281	1.0515	−4.0533
